# Inversed Cation Size Effects on Methanol Formations From CO_2_ Electroreduction by Immobilized Cobalt Phthalocyanine

**DOI:** 10.1002/anie.1450878

**Published:** 2026-05-18

**Authors:** Ke Ye, Min Hu, Guozhen Zhang, Mårten S. G. Ahlquist

**Affiliations:** ^1^ Division of Theoretical Chemistry and Biology KTH Royal Institute of Technology Stockholm Sweden; ^2^ School of Arts and Sciences Fuyao University of Science and Technology Fuzhou China; ^3^ Hefei National Research Center for Physical Sciences At the Microscale School of Chemistry and Materials Science University of Science and Technology of China Hefei China

**Keywords:** cation effects, CoPc, electrocatalysis CO_2_RR, methanol

## Abstract

The electrocatalytic reduction of CO_2_ to methanol offers a compelling pathway for sustainable fuel synthesis, wherein cations in the electric double layer (EDL) exert a substantial influence on catalytic performance. Although cation modulation of CO_2_‐to‐CO conversion has been extensively documented, its influence on downstream reduction pathways toward CH_3_OH has received comparatively little attention. Using multiscale simulation, we establish that methanol synthesis over immobilized cobalt phthalocyanine (CoPc) is kinetically governed by the final proton transfer (*CH_2_OH + H_2_O → * + CH_3_OH + OH^−^). The EDL environment substantially accelerates this rate‐determining step (RDS). Moreover, the activity exhibits a clear dependence on cation radius, following the trend Li^+^ > Na^+^ > K^+^ > Cs^+^, with smaller cations systematically lowering the proton transfer barrier. This trend stems from the enhanced accessibility of smaller cations to the transition state, where Li^+^ achieves tighter coordination than Cs^+^, conferring greater electrostatic stabilization and a correspondingly reduced barrier. Conversely, smaller cations attenuate the hydrogen‐bond network surrounding OH^−^, potentially impeding OH^−^ transfer from the catalyst surface to the bulk electrolyte. These multifaceted cation effects underscore the complex interplay between kinetic promotion and mass transfer limitations in electrocatalytic systems.

## Graphical abstract

Multiscale simulations reveal an inversed cation size effect (Li^+^ > Na^+^ > K^+^ > Cs^+^) in immobilized cobalt phthalocyanine ‐catalyzed CO_2_ reduction to methanol. Smaller cations like Li^+^ effectively stabilize the rate‐limiting proton‐transfer transition state through intimate coordination, whereas their disruption of the local hydrogen‐bond network highlights a delicate balance between kinetic promotion and mass‐transport limitations.

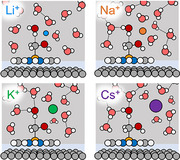

Cations residing in the EDL exert substantial influence over diverse electrocatalytic reductions, including the hydrogen evolution reaction [1, 2, 3, 4, 5, 6], oxygen reduction reaction [7, 8, 9, 10, 11, 12, 13], nitrogen reduction reaction [14, 15, 16, 17, 18], and CO_2_ reduction reaction (CO_2_RR) [19, 20, 21, 22, 23, 24, 25, 26, 27, 28, 29]. In CO_2_RR, a robust consensus has emerged that alkali metal cations in the EDL enhance CO_2_ chemisorption, though the magnitude of this effect varies with cation species [28, 29, 30]. Alkali metal cations exhibit a pronounced size‐activity correlation, with CO_2_RR performance ascending in the order Li^+^ < Na^+^ < K^+^ < Cs^+^ [30]. The origin of this size‐dependent activity has been the subject of considerable debate. One prevailing view posits that larger cations have lower dehydration energies, making them easier to dehydrate and thus more likely to coordinate with anionic intermediates such as *COO^−^, thereby promoting CO_2_ chemisorption [30]. Alternatively, larger cations have been proposed to augment interfacial electric fields (EF), creating a more favorable electrostatic environment for CO_2_ activation [31, 32, 33, 34]. Our previous work demonstrated that this activity ordering reflects cation‐driven anion redistribution within the EDL, where the interplay between cationic and anionic distributions collectively modulates catalytic activity [29]. Recent findings indicate that cation modulation is not confined to CO_2_ adsorption but permeates the entire multi‐electron reduction sequence [28, 35]. For instance, Ahlquist and co‐workers established that the EDL modulates not only the CO_2_ adsorption but also the sequential protonation pathway from *COO to *CO and the subsequent CO desorption [28]. However, mechanistic understanding of cation modulation in deep CO_2_ reduction—particularly toward energy‐dense liquid products such as methanol—remains underdeveloped.

Methanol production via CO_2_RR represents an especially complex scenario involving multiple proton‐coupled electron transfers (PCET). CoPc immobilized on carbon nanotubes(CoPc/CNT)—the only molecular catalyst capable of selective CO_2_‐to‐CH_3_OH conversion—provides a unique opportunity to probe cation effects in deep reduction pathways [26, 36, 37, 38, 39, 40, 41]. Notably, its catalytic performance exhibits strong cation sensitivity, though the atomic‐scale origins of this dependence are poorly understood [26, 37]. Shao‐Horn and co‐workers identified a Li^+^ > Na^+^ > K^+^ > Cs^+^ activity trend and proposed that the RDS is proton transfer during the *CHO → *CH_2_O transformation, with the higher acidity of hydrated Li^+^ promoting proton availability [37]. In contrast, Baker and co‐workers reported an opposite trend in which K^+^ outperforms Li^+^ for CO_2_‐to‐CH_3_OH conversion on CoPc/CNT [26]. By replotting the partial current densities from Shao–Horn and Baker's groups, Ringer revealed that the cation activity trend is potential‐dependent [42]: at low overpotentials, the partial current for CH_3_OH increases rapidly with increasing overpotential and the cation trend follows Li^+^ > K^+^; as the overpotential increases further the activity differences diminish. At higher overpotentials, the partial current for CH_3_OH approaches a plateau, which might be related to mass‐transport limitations, and the cation trend reverses to K^+^ > Li^+^. This suggests that proton transfer is likely RDS at low overpotentials, while mass transport becomes RDS at higher overpotentials. While this analysis provides a plausible explanation for the observed reversal in cation activity trend, the mechanistic origin of this potential‐dependent cation activity trend remains poorly understood, limiting systematic catalyst‐electrolyte design strategies.

In this study, we leverage an integrated density functional theory (DFT) and classical molecular dynamics (MD) approach to investigate the influence of cation identity on CO_2_RR over immobilized CoPc catalysts. We elucidate the energetics and molecular mechanisms of the CH_2_O to CH_3_OH conversion and identify the RDS. Furthermore, based on MD simulations, we uncover the fundamental mechanisms governing the experimentally observed correlation between cation radius and catalytic activity. These investigations illuminate the intricate balance between cation‐mediated kinetic enhancement and mass transfer inhibition, thereby providing theoretical guidance for the rational design of electrolyte composition in CO_2_RR.

We first examined the CH_2_O adsorption on CoPc and found that explicit water molecules are crucial for CH_2_O chemisorption on CoPc. Figure 1a shows that the Co─C(*CH_2_O) distance decreases progressively with increasing number of explicit water molecules in an implicit solvent environment. Without explicit water molecules, CH_2_O does not bind to the Co center with a Co─C distance of 3.3 Å. With one explicit water molecule, the Co─C distance reduces to 2.4 Å, and two explicit water molecules further shorten it to 2.1 Å. Upon adsorption, two hydrogen bonds between the explicit water molecules and *CH_2_O stabilize the *CH_2_O intermediate. Further increasing the number of explicit water molecules from 2 to 8 reduces the Co─C bond length from 2.1 to 2.0 Å. The alternative adsorption of CH_2_O to CoPc via the oxygen atom (*OCH_2_) was also examined. As shown in Figure 1b, with 8 explicit water molecules, *OCH_2_ configuration is 8.9 kcal mol^−1^ higher in energy than *CH_2_O configuration, indicating that *CH_2_O is thermodynamically more favorable in aqueous solution. CH_2_O is therefore expected to remain C‐bound throughout the CO_2_‐to‐CH_3_OH conversion, with the corresponding Gibbs free energy profile for *CH_2_O to CH_3_OH shown in Figure 1c. The final proton‐transfer step (*CH_2_OH + H_2_O → * + CH_3_OH) is identified as the RDS, consistent with experimental observations that proton is involved in the RDS [42, 43]. Actually, which of these PCET steps is the RDS for methanol production remains a topic of ongoing discussion [37, 38, 42, 43]. Although *CO‐to‐*CHO [38] and *CHO‐to‐*CH_2_O [37] have been widely proposed as the RDS. Ringe cautioned that Tafel slope analysis and kinetic isotope effects are often difficult to interpret due to mass transport effects and negligible isotope effects in PCET steps [44]. In contrast, pH‐dependent experiments are more informative and point to a rate‐limiting PCET step [42, 43]. The overall mechanism for CoPc‐catalyzed CO_2_RR to CH_3_OH is illustrated in Figure 1d.

**FIGURE 1:**
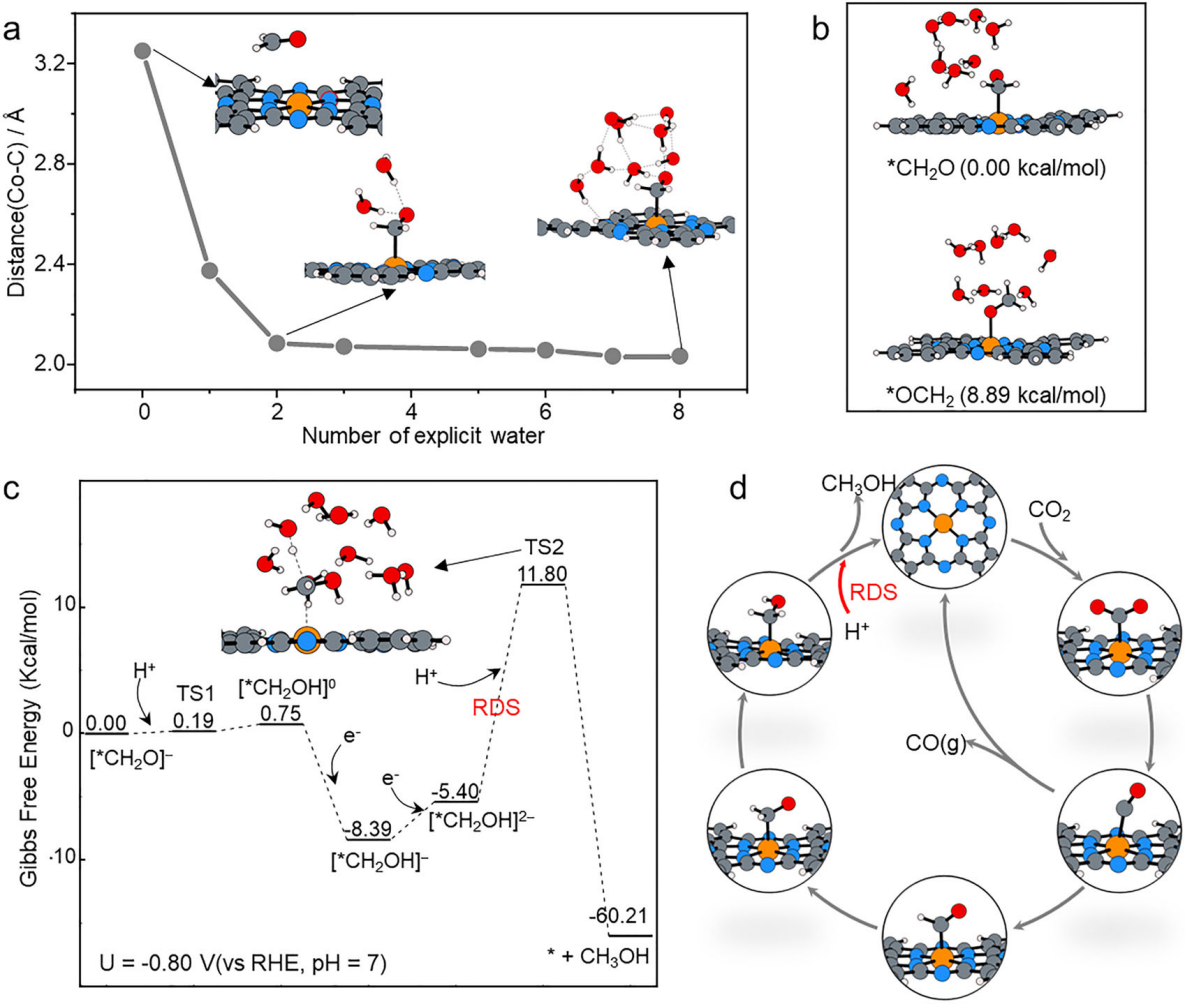
(a) Co─C(CH_2_O) bond length as a function of explicit water molecules under implicit solvation. (b) C‐bound and O‐bound adsorption configurations of CH_2_O with relative energies. (c) Reaction mechanism and free energy profile for CH_2_O‐to‐CH_3_OH conversion on CoPc. (d) An overall CO_2_‐to‐CH_3_OH mechanism on CoPc, with final protonation identified as the RDS.

To elucidate the size effect of cation within the EDL on CoPc‐catalyzed CO_2_‐to‐CH_3_OH conversion, MD‐based free energy perturbation (FEP) was performed to evaluate the barrier for the RDS. Figure 2a displays the top and side views of the CoPc catalyst, which is immobilized on graphene as shown in Figure 2b. Graphene is used as an approximation for CNT since the size of CNT used in experiments is significantly larger than CoPc, and CoPc is immobilized on the outer wall of the CNT via π–π interaction. While curvature of CNTs with a small diameter may slightly perturb the electronic structure of CoPc, it is not expected to affect the cation activity trend since the only variable in the FEP simulations is the cation force‐field parameters. The MD model comprises approximately 32,000 atoms within a 64 × 68 × 69 Å rectangular cell, containing an aqueous electrolyte solution with ∼0.5 mol L^−1^ XHCO_3_ (X = Li, Na, K, Cs). The MD simulations were conducted under an EF of −0.5 V nm^−1^ to effectively mimic the experimental overpotential for CO_2_‐to‐CH_3_OH conversion, consistent with our earlier work [45]. Figure 2c displays the initial state and transition state (TS) structures used in FEP calculations, which were obtained from DFT geometry optimizations. Based on FEP simulation, we obtained the energy barriers for the RDS in various cation environments, as illustrated in Figure 2d. The energy barrier of the RDS increases progressively with the cation radius increases. The methanol production activity follows the trend: Li^+^ > Na^+^ > K^+^ > Cs^+^, in complete agreement with experimental observation [37]. Across the four FEP simulations for Li^+^, Na^+^, K^+^, and Cs^+^, the only difference in force field parameters is the cation itself; any systematic errors in FEP are primarily attributable to the cation force field parameters. To minimize statistical errors, the Δ*G* ^≠^ reported in Figure 4d is the average of three independent FEP simulations for each cation (individual FEP results shown in Table S1).

**FIGURE 2:**
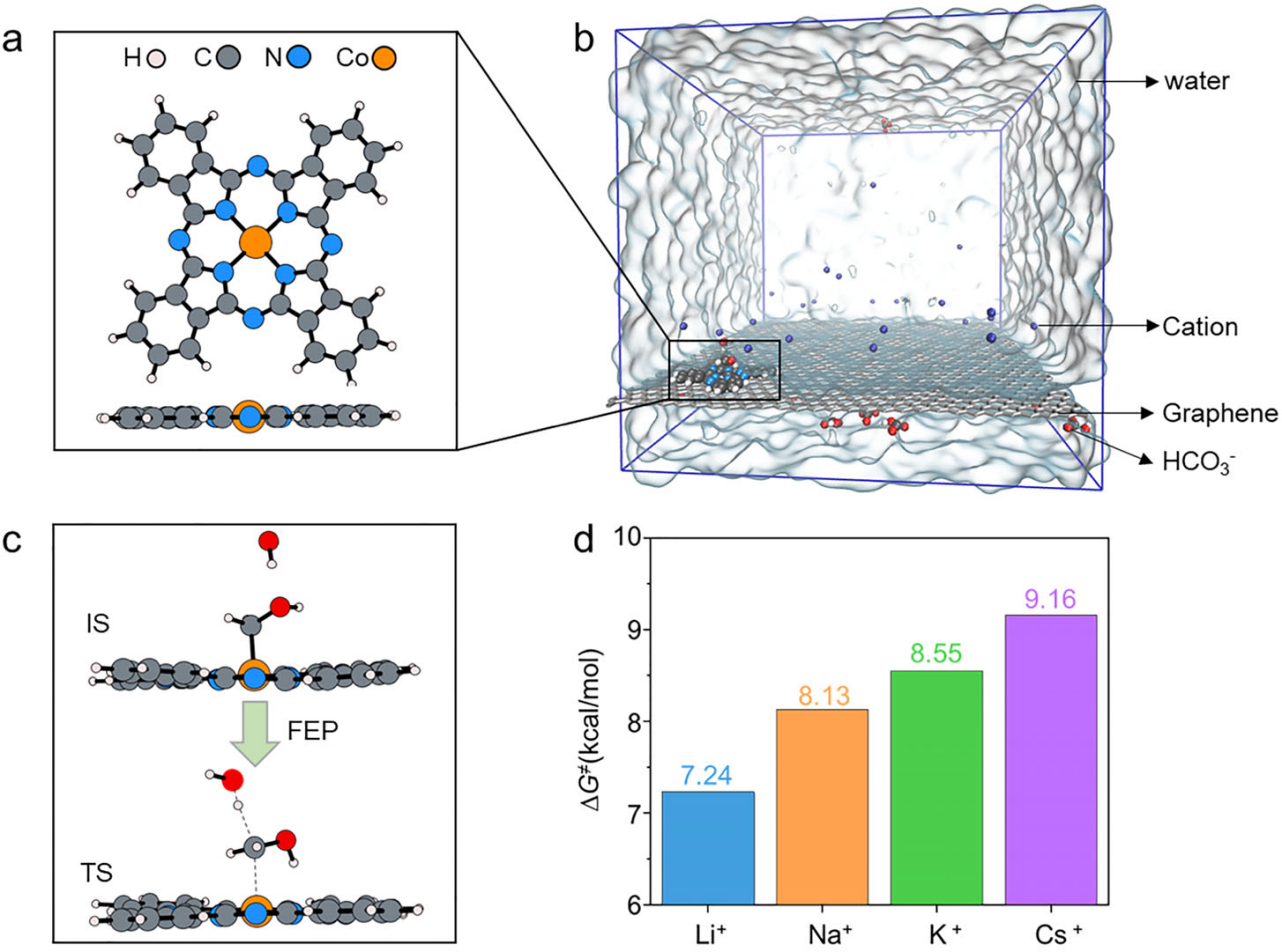
(a) Top and side views of the CoPc catalyst. (b) MD model employed for FEP simulations. (c) Initial state and transition state structures for FEP simulation of *CH_2_OH protonation to CH_3_OH on CoPc. (d) Proton transfer barriers in the presence of different alkali metal cations (Li^+^ to Cs^+^).

To elucidate the mechanism underlying this cation size effect, we analyzed the radial distribution function (RDF) of cations around the electronegative oxygen atoms (O1‐O2, Figure 3a) in the TS structure. As shown in Figure 3a, the primary RDF peak for Li^+^ is centered at 0.2 nm, with a maximum intensity of 1121. The coordination number (CN) of Li^+^ around O1‐O2 is 1.03. This strong coordination stabilizes the TS and lowers the energy barrier. Figure 3b–d shows the RDF curves and CN of Na^+^, K^+^, and Cs^+^. As the cation radius increases from Li^+^ to Cs^+^, the primary RDF peaks shift progressively from 0.20 to 0.24, 0.28, and 0.34 nm, respectively, reflecting increasing steric hindrance that prevents larger cations from closely approaching the O1‐O2. Representative MD snapshots in Figure 3e further confirm the progressive increase in cation‐oxygen(O1‐O2) distances from Li^+^ to Cs^+^.

**FIGURE 3:**
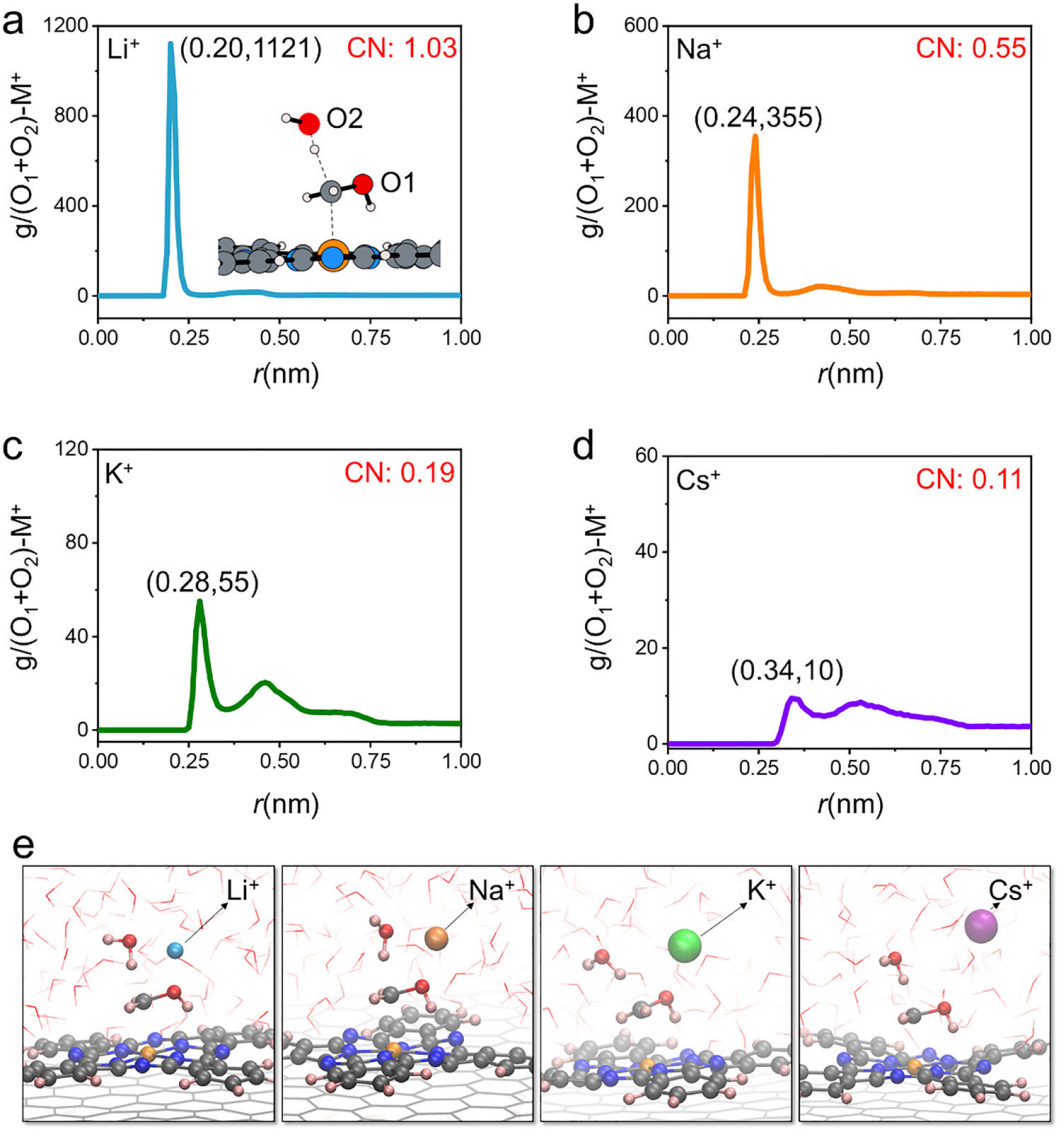
(a–d) Radial distribution functions (RDFs) of cations around the two electronegative O atoms in the transition state of *CH_2_OH protonation: (a) Li^+^, (b) Na^+^, (c) K^+^, (d) Cs^+^. Values in parentheses indicate the first peak position, and red numbers denote coordination numbers. (e) Representative snapshots from MD trajectories.

Notably, the intensities of the first RDF peak drop sharply from 1121 (Li^+^) to 355 (Na^+^), 55 (K^+^), and 10 (Cs^+^), indicating progressively weaker cation coordination with O1‐O2 as the cation radius increases. This is further corroborated by the CN values, decreasing progressively from 1.03(Li^+^) to 0.55(Na^+^), 0.19(K^+^), and 0.11(Cs^+^). Collectively, these results reveal a size‐dependent stabilization mechanism in which the coordinating ability of cation with O1‐O2 decreases progressively from Li^+^ to Cs^+^, giving a coordination strength trend: Li^+^ > Na^+^ > K^+^ > Cs^+^, with smaller cations more effectively stabilizing the TS and promoting the reaction. This coordination trend contrasts with the conventional view that the cation coordination trend (Cs^+^ > K^+^ > Na^+^ > Li^+^) is governed by dehydration energy. For example, in CO_2_ adsorption, the lower dehydration penalty of larger cations such as Cs^+^ facilitates their coordination with and stabilization of the electronegative *COO^−^ intermediate [30]. Based on previous studies, we propose the following mechanism for this discrepancy.

Although Li^+^ has the highest dehydration energy and is therefore hardest to partially dehydrate, once it is partially dehydrated, its small size and high charge density enable the strongest coordination with electronegative intermediates. For partially hydrated cations, both rehydration with water and the coordination with electronegative intermediates follow the same trend (Li^+^ > Na^+^ > K^+^ > Cs^+^). The trend of cation coordinating with electronegative intermediates thus depends on the competition between two processes: (1) partially hydrated cation rehydration with water, and (2) partially hydrated cation coordination with the electronegative intermediate. When process (2) is more favorable, it dominates the trend of cation coordinating with electronegative intermediates, leading to Li^+^ > Na^+^ > K^+^ > Cs^+^. In this regime, CN are relatively large, due to the partially hydrated cations preferentially coordinating with the intermediate. This is the case in our work, where the CN for Li^+^, Na^+^, K^+^, and Cs^+^ are 1.03, 0.55, 0.19, and 0.11, respectively. Conversely, when process (2) is less favorable, the trend of cation coordinating with electronegative intermediates is dominated by process (1), giving Cs^+^ > K^+^ > Na^+^ > Li^+^. In this regime, the CN are relatively small, as the cations tend to rehydrate. This is the case in our previous work on CO_2_ adsorption, which reported CN of 0.015, 0.026, 0.047, and 0.037 for Li^+^, Na^+^, K^+^, and Cs^+^, respectively [29].

Usually, a higher negative charge density on the intermediate will favor process (2). This is supported by Koper and co‐workers, who reported a Cs^+^ > K^+^ > Na^+^ > Li^+^ trend for *COO^−^ on Cu, where the charge of *COO^−^ is ∼−0.6 |e^–^| [30]. Our previous work on CO_2_ adsorption on Ni‐N‐C catalysts also showed relatively modest charge on *COO^–^(−0.538 and −0.537 on the two O atoms) [29]. In contrast, the two oxygen atoms in this work carry more negative charges (−0.71 and −0.95 |e^–^| on O1 and O2, Figure 3a), leading to a reversed trend of Li^+^ > Na^+^ > K^+^ > Cs^+^.

After the proton transfer (*CH_2_OH + H_2_O → * + CH_3_OH), OH^−^ will migrate into the bulk solution via the Grotthuss mechanism. Since this mass transfer process is intrinsically linked to the hydrogen‐bond network [5], we examined the local hydrogen‐bond network within 5 Å of OH^−^ (Figure 4a). Figure 4b shows the RDF of hydrogen in H_2_O around oxygen in H_2_O. The RDF peak positions remain invariant across four cations, while the peak intensity increases with cation size. This suggests that Cs^+^ promotes stronger hydrogen‐bond network connectivity around OH^−^, whereas Li^+^ induces weaker hydrogen‐bond connectivity. Since an enhanced hydrogen‐bond network facilitates proton transport [5]. It can be qualitatively inferred that mass transport is likely less efficient in the Li^+^ solution than in the Cs^+^ solution. This is consistent with the experimental observation that, in Li^+^ solution, the partial currents for CH_3_OH and H_2_ reach a plateau at lower overpotentials.

**FIGURE 4:**
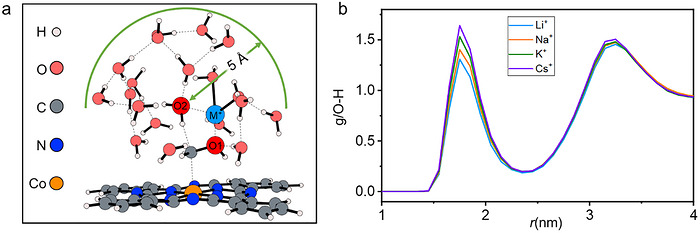
(a) Transition state structure for *CH_2_OH protonation on CoPc, highlighting water molecules within 5 Å of the OH^−^ product. (b) RDF of H atoms around O atoms in the 5 Å solvation shell of OH^−^. The first peak intensity decreases in the order Cs^+^ > K^+^ > Na^+^ > Li^+^, indicating weaker hydrogen‐bond networks with smaller cations.

As illustrated in Figure 5, the cation size effect in CoPc‐catalyzed CO_2_ to CH_3_OH manifests in two mechanisms. First, Li^+^ stabilizes the TS more effectively through stronger coordination with O1‐O2, thereby reducing the energy barrier for the proton transfer step. Conversely, Li^+^ induces weaker hydrogen‐bond connectivity, which leads to mass‐transport limitations. The multifaceted nature of cations accounts for the experimentally observed potential‐dependent cation trend in CO_2_‐to‐CH_3_OH conversion on CoPc/CNT [42]. At low overpotentials, the RDS is likely proton transfer; the catalytic activity of cations in the final protonation step follows the trend Li^+^ > K^+^. At high overpotentials, where the CH_3_OH partial current approaches a plateau, which might be related to mass‐transport limitations, the cation trend reverses to K^+^ > Li^+^.

**FIGURE 5:**
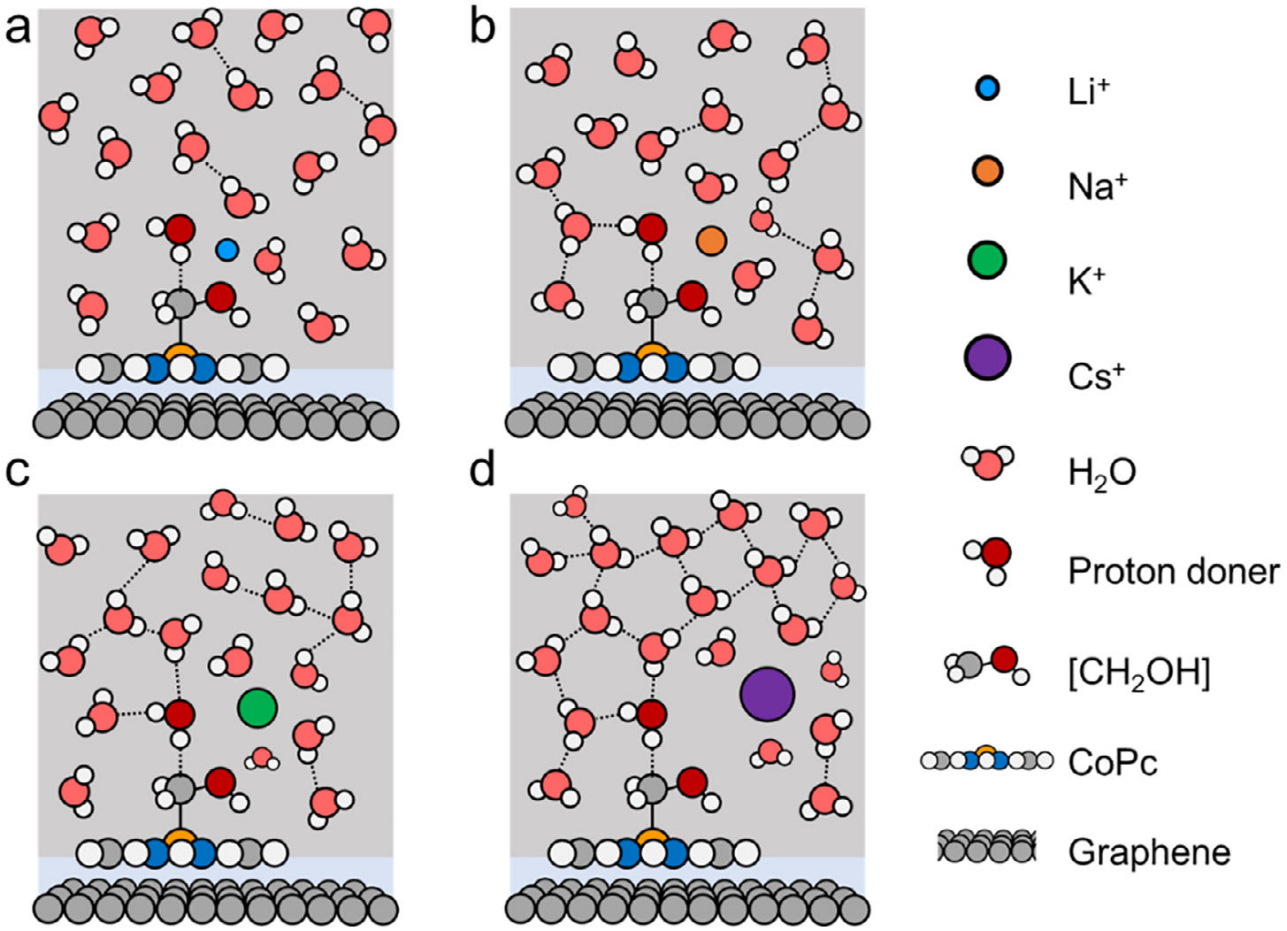
Schematic illustration of cation distribution and hydrogen‐bond network connectivity around OH^−^ at the transition state of the RDS *CH_2_OH protonation step in CoPc‐catalyzed CO_2_‐to‐methanol conversion.

In this work, we used multiscale simulations to investigate the effects of different cations on the key proton‐transfer step and hydrogen‐bond network connectivity. Our calculations show that the proton transfer barrier decreases systematically with decreasing cation radius: Li^+^ (7.24 kcal mol^−1^) < Na^+^ (8.13 kcal mol^−1^) < K^+^ (8.55 kcal mol^−1^) < Cs^+^ (9.16 kcal mol^−1^). This trend originates from the enhanced coordination effect of smaller Li^+^ to the electronegative O1‐O2 atoms in the TS structure, enabling stronger non‐covalent interactions that stabilize the TS and lower the barrier. Beyond kinetic promotion, we identify a competing effect: smaller cations weaken the hydrogen‐bond network around OH^−^, potentially hindering its diffusion from the catalyst surface into the bulk solution. These findings reveal the multifaceted regulatory role of interfacial cations in electrocatalysis, providing mechanistic insights for the rational design of electrocatalyst‐electrolyte systems.

## Author Contributions

**Ke Ye**: conceptualization, methodology, investigation, validation, formal analysis, writing–original draft, data curation, software. **Min Hu**: visualization, investigation, writing–original draft. **Mårten S. G. Ahlquist**: writing–original draft, supervision, validation, funding acquisition, resources, project administration, writing–review and editing. **Guozhen Zhang**: writing‐original draft, supervision, validation.

## Conflicts of Interest

The authors declare no conflicts of interest.

## Supporting information




**Supporting File 1**: anie72764‐sup‐0001‐SuppMat.docx.
All details of the computational study, including the DFT functional, basis sets, and software packages used, as well as the force field parametrization, molecular dynamics simulations, free energy perturbation, and the corresponding geometry file.

## Data Availability

The data that support the findings of this study are openly available in Zenodo at https://doi.org/10.5281/zenodo.18832745, reference number 18832745.
