# Supplementary material for: Inversed Cation Size Effects on Methanol Formations From CO2 Electroreduction by Immobilized Cobalt Phthalocyanine
Source: Angew Chem Int Ed Engl. 2026 May 18;65(27):e1450878. doi: 10.1002/anie.1450878 (PMC13327561; doi:10.1002/anie.1450878)
Supplement: Supplementary file 1 — Supporting File 1: anie72764‐sup‐0001‐SuppMat.docx. All details of the computational study, including the DFT functional, basis sets, and software packages used, as well as the force field parametrization, molecular dynamics simulations, free energy perturbation, and the corresponding geometry file. [file ANIE-65-e1450878-s001.docx]

# Supplementary Information

**Inversed Cation Size Effects on Methanol Formations from CO_2_ Electroreduction by Immobilized Cobalt Phthalocyanine**

Ke Ye^[a]^, Min Hu^[b]^, Guozhen Zhang^[c]^, Mårten S. G. Ahlquist*^[a]^

[a] Division of Theoretical Chemistry and Biology, KTH Royal Institute of Technology, 10691Stockholm, Sweden Email: [ahlqui@kth.se](mailto:ahlqui@kth.se)

[b] School of Arts and Sciences, Fuyao University of Science and Technology, Fuzhou, China

[c] Hefei National Research Center for Physical Sciences at the Microscale, School of Chemistry and Materials Science, University of Science and Technology of China, Hefei 230026, China.

Contents

[Supplementary Information 1](#_Toc225843437)

[1. Computational Detail 3](#_Toc225843438)

[1.1 DFT calculation 3](#_Toc225843439)

[1.2 MD simulation 3](#_Toc225843440)

[2. DFT and MD simulation results 11](#_Toc225843441)

[3. Reference 13](#_Toc225843442)

## Computational Detail

### 1.1 DFT calculation

All DFT calculations were executed using the Gaussian16 package^[1]^. The calculations were executed using B3LYP-D3 density functional^[2]^ with solvation effects modeled via the SMD continuum approach^[3]^. For geometry optimizations, we employed a mixed basis set: the SDD^[4]^ was used for the Co atom, and all the other elements were treated with 6-31G(d,p) basis set. Vibrational frequency calculations were performed at the same theoretical level to confirm the nature of the stationary point and to obtain the Gibbs free energy corrections at 298.15 K. To enhance the accuracy of electronic energies, we performed subsequent single-point calculations with an expanded basis set (6-311+G(2df,2p)) for all atoms except cobalt, which retained the SDD treatment. For electrochemical calculations, we referenced all potentials to the standard hydrogen electrode (SHE) using its established absolute potential of 4.281 V^[5]^ (corresponding to an electron affinity of 98.7 kcal/mol^[6]^). Unless otherwise noted, all the redox potentials reported in the present work are relative to RHE (pH = 7). The proton's thermodynamic parameters were incorporated using its gas-phase Gibbs free energy (-6.3 kcal/mol) and aqueous solvation free energy (-264.0 kcal/mol^[6]^), yielding a total aqueous-phase Gibbs free energy of -270.3 kcal/mol. The standard state corrections were implemented as follows: a 1.9 kcal/mol adjustment was applied to most species to account for the concentration change from ideal gas (1 atm) to aqueous solution (1 M).

### 1.2 MD simulation

All Molecular Dynamics (MD) simulations were performed using the GROMACS 2022.2 package^[7]^. Restrained electrostatic potential (RESP) charges were computed to partition the electron by Multiwfn software^[8]^. Forcefield parameters are based on the OPLS-AA force field^[9]^. The equilibrium bond lengths, angles, and dihedrals were taken from the optimized structures performed by Gaussian16. Sobtop software^[10]^ was used to help build the parameters that were still missing. The TIP/3P model was used because of its superior ability to replicate the experimental dielectric constant of water compared to other widely used water models^[11]^. To avoid self-interactions under periodic boundary conditions, a sufficiently large box of 64 × 68 × 69 Å^3^ was used for all simulations. We used a graphene sheet to model the interaction between the CoPc and the carbon nanotubes since large multi-walled nanotubes were used in the experiments. The diameter of the CNT is much larger than the size of a CoPc molecule, rendering the local surface on which CoPc is adsorbed effectively planar. Such an approximation has also been adopted in previous theoretical studies^[12]^, where it was confirmed that modeling CoPc immobilized on CNTs using CoPc on graphene yields results largely insensitive to the strain effects expected for large-diameter CNTs. The curvature of the small-diameter CNT and catalyst-support heterogeneity may influence the adsorption of intermediates such as *CO and the associated charge distribution, which could introduce an overall shift in the proton transfer barrier for the final step. However, since the only variable across the four FEP simulations is the cation force field parameters, such effects would shift all four cation barriers equally and would not qualitatively affect the relative cation activity trend. Local confinement effects may influence mass transport processes and thereby affect the local concentrations of CO2 and CO near CoPc, as well as other dynamic transport phenomena, but are expected to have a minimal effect on the cation coordination interactions. Following our previous work^[13]^, to accurately describe the non-covalent interactions between graphene and CoPc, we increased the epsilon value of the graphene carbon atoms, as shown below:

[ atomtypes ]

; name mass charge ptype sigma eps

ca 6 12.010736 0.000000 A 3.550000E-01 5.85760E-01
While the standard is:
[ atomtypes ]

; name mass charge ptype sigma eps

ca 6 12.010736 0.000000 A 3.550000E-01 2.92880E-01

Further details of the FEP simulation input files are available at: <https://zenodo.org/records/17098658>

#### 1.2.1 .mdp file for FEP simulation in water:

title = OPLS Lysozyme NPT equilibration

; Run parameters

integrator = md ; leap-frog integrator

nsteps = 2000000 ; 1 * 100000000 = 100000 ps (100 ns)

dt = 0.001 ; 1 fs

; Output control

nstxout = 100000 ; suppress bulky .trr file by specifying

nstvout = 100000 ; 0 for output frequency of nstxout,

nstfout = 100000 ; nstvout, and nstfout

nstenergy = 200 ; save energies every 10.0 ps

nstlog = 100000 ; update log file every 10.0 ps

nstxout-compressed = 2000 ; save compressed coordinates every 10.0 ps

compressed-x-grps = System ; save the whole system

; Bond parameters

continuation = yes ; Restarting after NPT

constraint_algorithm = lincs ; holonomic constraints

constraints = h-bonds ; bonds involving H are constrained

lincs_iter = 1 ; accuracy of LINCS

lincs_order = 4 ; also related to accuracy

; Neighborsearching

cutoff-scheme = Verlet ; Buffered neighbor searching

ns_type = grid ; search neighboring grid cells

nstlist = 10 ; 20 fs, largely irrelevant with Verlet scheme

rcoulomb = 1.0 ; short-range electrostatic cutoff (in nm)

rvdw = 1.0 ; short-range van der Waals cutoff (in nm)

; Electrostatics

coulombtype = PME ; Particle Mesh Ewald for long-range electrostatics

pme_order = 4 ; cubic interpolation

fourierspacing = 0.16 ; grid spacing for FFT

; Temperature coupling is on

tcoupl = V-rescale ; modified Berendsen thermostat

tc-grps = system ; two coupling groups - more accurate

tau_t = 0.1 ; time constant, in ps

ref_t = 300 ; reference temperature, one for each group, in K

Pressure coupling is on

pcoupl = Berendsen ; Pressure coupling on in NPT

pcoupltype = isotropic ; uniform scaling of box vectors

tau_p = 2.0 ; time constant, in ps

ref_p = 1.0 ; reference pressure, in bar

compressibility = 4.5e-5 ; isothermal compressibility of water, bar^-1

refcoord_scaling = com

;ref_p = 1.0 ; reference pressure, in bar

;compressibility = 4.5e-5 ; isothermal compressibility of water, bar^-1

; Periodic boundary conditions

pbc = xyz ; 3-D PBC

; Dispersion correction

DispCorr = EnerPres ; account for cut-off vdW scheme

; Velocity generation

gen_vel = no ; Velocity generation is off

;define = -DPOSRES ; position restrain the graphene

;electric-field-z = -0.5 0 0 0; Electric field on z with a value in V/nm

; Free energy control parameters

free_energy = yes

init_lambda_state = 0

delta_lambda = 0

calc_lambda_neighbors = 1

couple-lambda0 = vdw-q

couple-lambda1 = vdw-q

couple-intramol = yes

; Vectors of lambda specified here

; init_lambda_state 0 1 2 3 4 5 6 7 8 9 10

vdw_lambdas = 0.00 0.00 0.00 0.00 0.00 0.00 0.00 0.00 0.00 0.00 0.00 0.00 0.00 0.00 0.00 0.00 0.00 0.00 0.00 0.00 0.00

coul_lambdas = 0.00 .0500 .1000 .1500 .2000 .2500 .3000 .3500 .4000 .4500 .5000 .5500 .6000 .6500 .7000 .7500 .8000 .8500 .9000 .9500 1.0000

bonded_lambdas = 0.00 .0500 .1000 .1500 .2000 .2500 .3000 .3500 .4000 .4500 .5000 .5500 .6000 .6500 .7000 .7500 .8000 .8500 .9000 .9500 1.0000

restraint_lambdas = 0.00 .0500 .1000 .1500 .2000 .2500 .3000 .3500 .4000 .4500 .5000 .5500 .6000 .6500 .7000 .7500 .8000 .8500 .9000 .9500 1.0000

; Masses are not changing (particle identities are the same at lambda = 0 and lambda = 1)

mass_lambdas = 0.00 0.00 0.00 0.00 0.00 0.00 0.00 0.00 0.00 0.00 0.00 0.00 0.00 0.00 0.00 0.00 0.00 0.00 0.00 0.00 0.00

; Not doing simulated temperting here

temperature_lambdas = 0.00 0.00 0.00 0.00 0.00 0.00 0.00 0.00 0.00 0.00 0.00 0.00 0.00 0.00 0.00 0.00 0.00 0.00 0.00 0.00 0.00

; Options for the decoupling

sc-alpha = 0.5

sc-coul = no ; linear interpolation of Coulomb (none in this case)

sc-power = 1

sc-sigma = 0.3

nstdhdl = 10

disre = simple

nstdisreout = 0

#### 1.2.2 .mdp file for FEP simulation in EDL:

title = OPLS Lysozyme NPT equilibration

; Run parameters

integrator = md ; leap-frog integrator

nsteps = 2000000 ; 1 * 100000000 = 100000 ps (100 ns)

dt = 0.001 ; 1 fs

; Output control

nstxout = 100000 ; suppress bulky .trr file by specifying

nstvout = 100000 ; 0 for output frequency of nstxout,

nstfout = 100000 ; nstvout, and nstfout

nstenergy = 200 ; save energies every 10.0 ps

nstlog = 100000 ; update log file every 10.0 ps

nstxout-compressed = 2000 ; save compressed coordinates every 10.0 ps

compressed-x-grps = System ; save the whole system

; Bond parameters

continuation = yes ; Restarting after NPT

constraint_algorithm = lincs ; holonomic constraints

constraints = h-bonds ; bonds involving H are constrained

lincs_iter = 1 ; accuracy of LINCS

lincs_order = 4 ; also related to accuracy

; Neighborsearching

cutoff-scheme = Verlet ; Buffered neighbor searching

ns_type = grid ; search neighboring grid cells

nstlist = 10 ; 20 fs, largely irrelevant with Verlet scheme

rcoulomb = 1.0 ; short-range electrostatic cutoff (in nm)

rvdw = 1.0 ; short-range van der Waals cutoff (in nm)

; Electrostatics

coulombtype = PME ; Particle Mesh Ewald for long-range electrostatics

pme_order = 4 ; cubic interpolation

fourierspacing = 0.16 ; grid spacing for FFT

; Temperature coupling is on

tcoupl = V-rescale ; modified Berendsen thermostat

tc-grps = system ; two coupling groups - more accurate

tau_t = 0.1 ; time constant, in ps

ref_t = 300 ; reference temperature, one for each group, in K

; Pressure coupling is on

pcoupl = Berendsen ; Pressure coupling on in NPT

pcoupltype = semiisotropic ; uniform scaling of box vectors

tau_p = 2.0 ; time constant, in ps

ref_p = 1.0 1.0 ; reference pressure, in bar

compressibility = 4.5e-5 4.5e-5 ; isothermal compressibility of water, bar^-1

refcoord_scaling = com

;ref_p = 1.0 ; reference pressure, in bar

;compressibility = 4.5e-5 ; isothermal compressibility of water, bar^-1

; Periodic boundary conditions

pbc = xyz ; 3-D PBC

; Dispersion correction

DispCorr = EnerPres ; account for cut-off vdW scheme

; Velocity generation

gen_vel = no ; Velocity generation is off

define = -DPOSRES ; position restrain the graphene

electric-field-z = -0.5 0 0 0; Electric field on z with a value in V/nm

; Free energy control parameters

free_energy = yes

init_lambda_state = 0

delta_lambda = 0

calc_lambda_neighbors = 1

couple-lambda0 = vdw-q

couple-lambda1 = vdw-q

couple-intramol = yes

; Vectors of lambda specified here

; init_lambda_state 0 1 2 3 4 5 6 7 8 9 10

vdw_lambdas = 0.00 0.00 0.00 0.00 0.00 0.00 0.00 0.00 0.00 0.00 0.00 0.00 0.00 0.00 0.00 0.00 0.00 0.00 0.00 0.00 0.00

coul_lambdas = 0.00 .0500 .1000 .1500 .2000 .2500 .3000 .3500 .4000 .4500 .5000 .5500 .6000 .6500 .7000 .7500 .8000 .8500 .9000 .9500 1.0000

bonded_lambdas = 0.00 .0500 .1000 .1500 .2000 .2500 .3000 .3500 .4000 .4500 .5000 .5500 .6000 .6500 .7000 .7500 .8000 .8500 .9000 .9500 1.0000

restraint_lambdas = 0.00 .0500 .1000 .1500 .2000 .2500 .3000 .3500 .4000 .4500 .5000 .5500 .6000 .6500 .7000 .7500 .8000 .8500 .9000 .9500 1.0000

; Masses are not changing (particle identities are the same at lambda = 0 and lambda = 1)

mass_lambdas = 0.00 0.00 0.00 0.00 0.00 0.00 0.00 0.00 0.00 0.00 0.00 0.00 0.00 0.00 0.00 0.00 0.00 0.00 0.00 0.00 0.00

; Not doing simulated temperting here

temperature_lambdas = 0.00 0.00 0.00 0.00 0.00 0.00 0.00 0.00 0.00 0.00 0.00 0.00 0.00 0.00 0.00 0.00 0.00 0.00 0.00 0.00 0.00

; Options for the decoupling

sc-alpha = 0.5

sc-coul = no ; linear interpolation of Coulomb (none in this case)

sc-power = 1

sc-sigma = 0.3

nstdhdl = 10

disre = simple

nstdisreout = 0

## DFT and MD simulation results


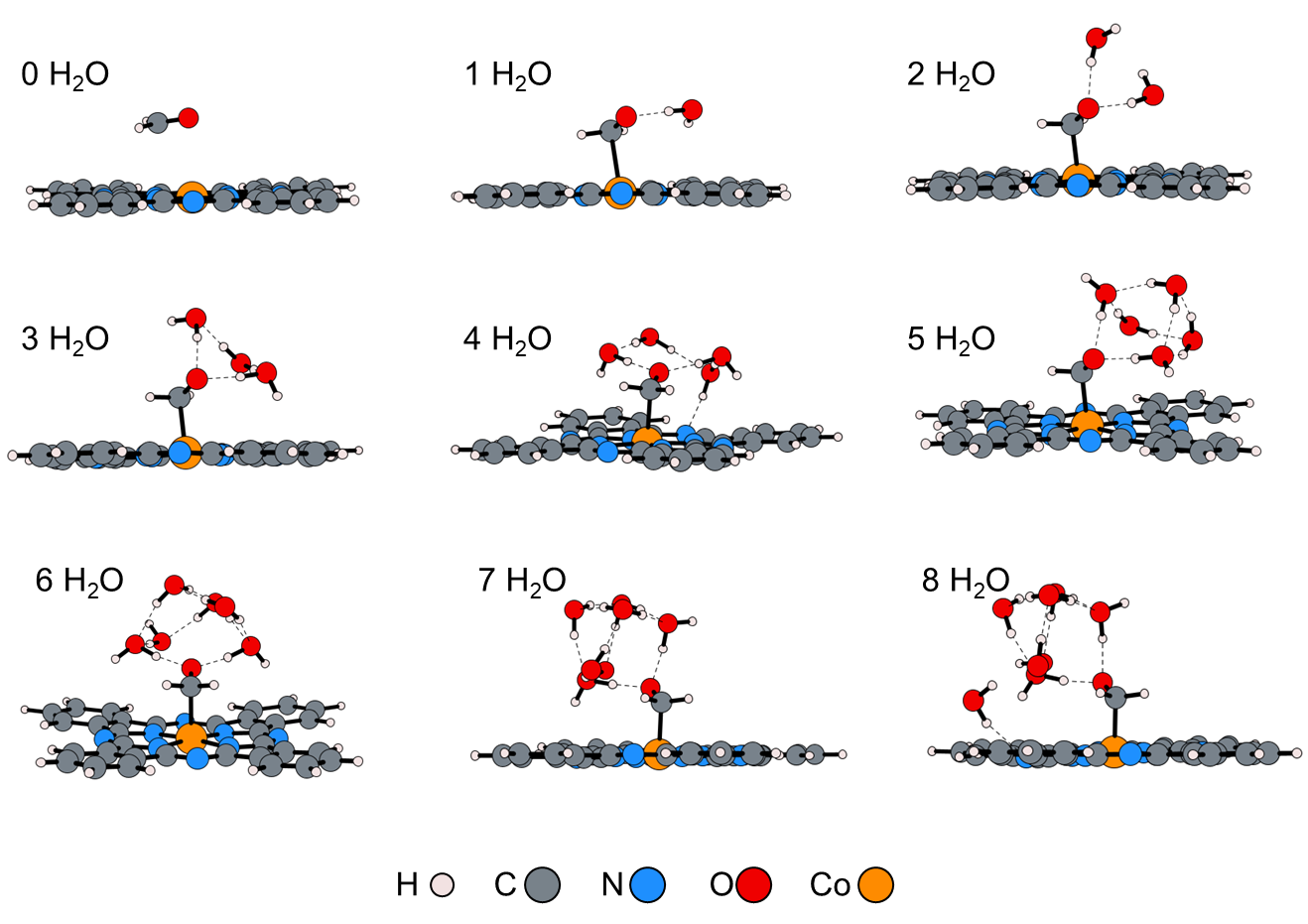


Figure S1. DFT-optimized adsorption configurations of CH_2_O on CoPc. As the number of explicit water molecules increases, the Co–C(CH_2_O) bond length gradually decreases. The hydrogen atoms of the explicit water molecules form two hydrogen bonds with the O atom of *CH_2_O, which significantly enhance the adsorption of *CH_2_O on CoPc. The dashed lines indicate hydrogen bonds.


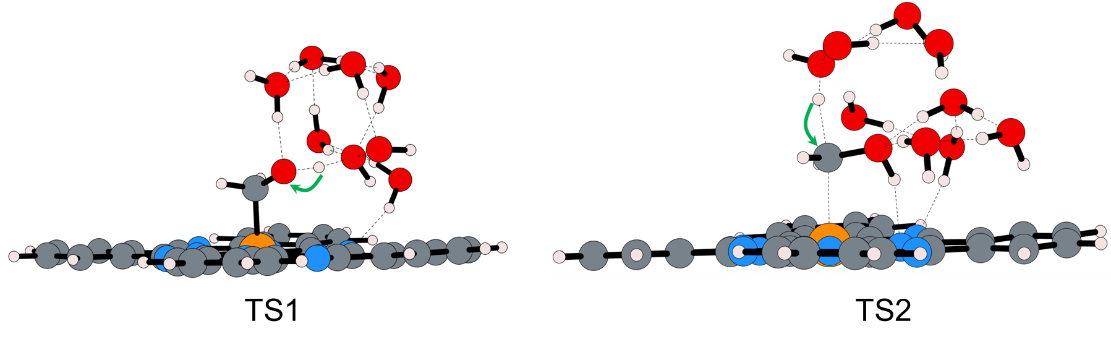


Figure S2. The transition-state structures for the two proton transfer steps during the conversion of *CH_2_O to CH_3_OH, where the green arrows indicate the proton transfer pathways.

Table S1. FEP results for the rate-determining proton transfer step (*CH_2_OH + H_2_O → * + CH_3_OH) in the presence of different alkali metal cations in the EDL, using the FEP result in pure water as the reference. The corresponding free energy barriers (∆*G*^≠^) are also listed. All values are given in kcal/mol.

|  | FEP1 | FEP2 | FEP3 | ∆*G*^≠^(*CH_2_OH + H_2_O → * + CH_3_OH) |
| --- | --- | --- | --- | --- |
| Li^+^ | -9.49 | -10.16 | -10.22 | 7.24 |
| Na^+^ | -8.82 | -8.81 | -9.56 | 8.13 |
| K^+^ | -8.78 | -8.26 | -8.89 | 8.55 |
| Cs^+^ | -8.02 | -7.70 | -8.38 | 9.16 |


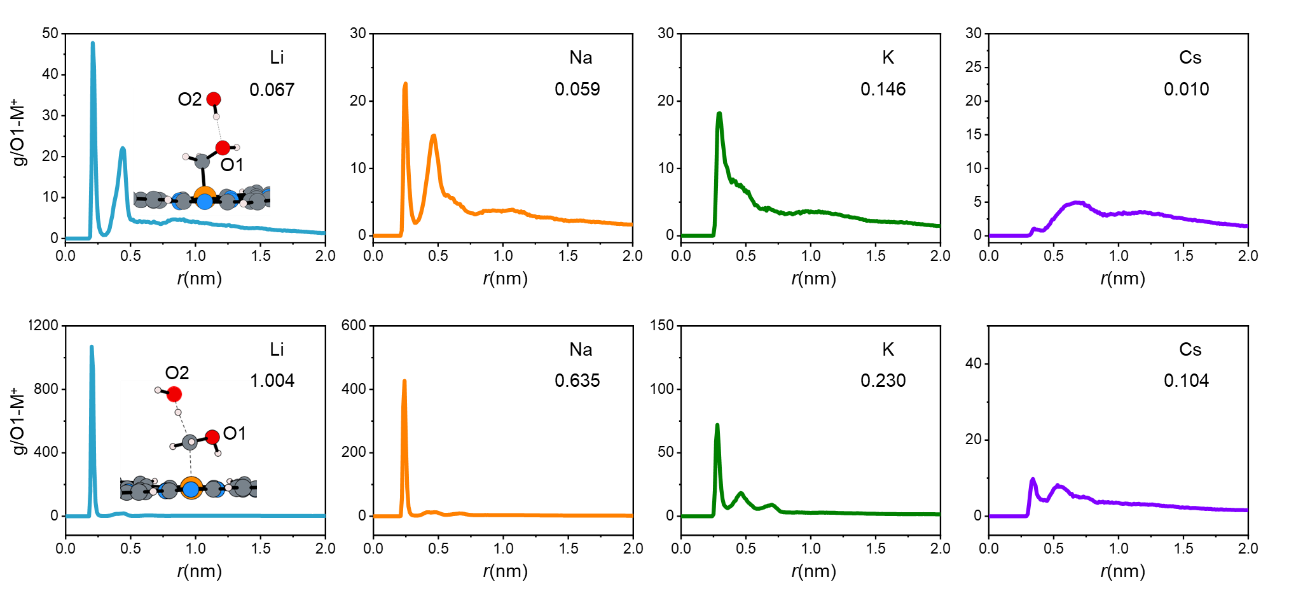


Figure S3. Radial distribution functions (RDFs) of cations around the electronegative O1 atom in *CH_2_OH in the initial state (top row) and transition state (bottom row) of the proton transfer process. The black numbers in the figure indicate the cation coordination numbers around O1.


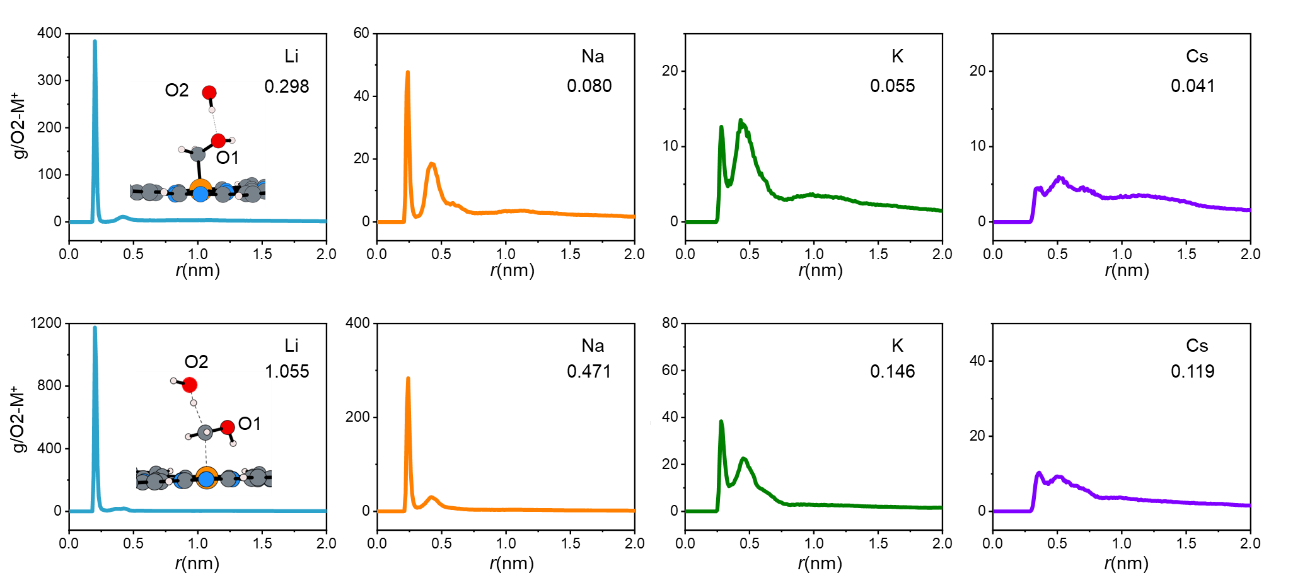


Figure S4. Radial distribution functions (RDFs) of cations around the electronegative O2 atom in H_2_O(proton donor) in the initial state (top row) and transition state (bottom row) of the proton transfer process. The black numbers in the figure indicate the cation coordination numbers around O2.

Table S2. The RDF peak heights and coordination numbers of H atoms around the O atoms of water molecules located within 5 Å of the H_2_O oxygen atom in the transition state of *CH_2_OH + H_2_O → * + CH_3_OH.

|  | Density of first RDF peak | Coordination number |
| --- | --- | --- |
| Li^+^ | 1.31 | 1.49 |
| Na^+^ | 1.40 | 1.61 |
| K^+^ | 1.53 | 1.72 |
| Cs^+^ | 1.64 | 1.81 |

More details of the FEP simulation input files and MD trajectories are available at: <https://zenodo.org/records/18832745> (DOI [10.5281/zenodo.18832744](https://doi.org/10.5281/zenodo.18832744))

## Reference

[1] M. Frisch, *Revision B* **2016**, *1*.

[2] aS. Grimme, J. Antony, S. Ehrlich, H. Krieg, *The Journal of chemical physics* **2010**, *132*; bA. Becke, *Chem. Phys*, *98*, 5648.

[3] A. V. Marenich, C. J. Cramer, D. G. Truhlar, *The Journal of Physical Chemistry B* **2009**, *113*, 6378-6396.

[4] D. Andrae, U. Haeussermann, M. Dolg, H. Stoll, H. Preuss, *Theoretica chimica acta* **1990**, *77*, 123-141.

[5] aC. J. Cramer, J. A. Bumpus, A. Lewis, C. Stotts, *Journal of chemical education* **2007**, *84*, 329; bD. G. Truhlar, C. J. Cramer, A. Lewis, J. A. Bumpus, *Journal of chemical education* **2004**, *81*, 596.

[6] A. A. Isse, A. Gennaro, *The Journal of Physical Chemistry B* **2010**, *114*, 7894-7899.

[7] aH. J. C. Berendsen, D. van der Spoel, R. van Drunen, *Computer Physics Communications* **1995**, *91*, 43-56; bM. J. Abraham, T. Murtola, R. Schulz, S. Páll, J. C. Smith, B. Hess, E. Lindahl, *SoftwareX* **2015**, *1*, 19-25.

[8] T. Lu, F. Chen, *Journal of Computational Chemistry* **2012**, *33*, 580-592.

[9] W. L. Jorgensen, D. S. Maxwell, J. Tirado-Rives, *Journal of the American Chemical Society* **1996**, *118*, 11225-11236.

[10] S. Tian Lu, Version [Version 1.0], <http://sobereva.com/soft/Sobtop> (accessed on Dec. 26, 2023).

[11] aW. L. Jorgensen, *ChemInform* **2002**, *12*; bJ.-P. Ryckaert, G. Ciccotti, H. J. C. Berendsen, *Journal of Computational Physics* **1977**, *23*, 327-341; cA. Alexiadis, S. Kassinos, *Chemical Reviews* **2008**, *108*, 5014-5034.

[12] P. Hutchison, L. E. Smith, C. L. Rooney, H. Wang, S. Hammes-Schiffer, *J Am Chem Soc* **2024**, *146*, 20230-20240.

[13] J. A. de Gracia Triviño, M. S. G. Ahlquist, *ACS Catalysis* **2023**, *13*, 1270-1279.
